# Anammox Bacteria Are Potentially Involved in Anaerobic Ammonium Oxidation Coupled to Iron(III) Reduction in the Wastewater Treatment System

**DOI:** 10.3389/fmicb.2021.717249

**Published:** 2021-09-10

**Authors:** Xiao-Ru Yang, Hu Li, Jian-Qiang Su, Guo-Wei Zhou

**Affiliations:** ^1^Key Lab of Urban Environment and Health, Institute of Urban Environment, Chinese Academy of Sciences (CAS), Xiamen, China; ^2^Center for Excellence in Regional Atmospheric Environment, Institute of Urban Environment, Chinese Academy of Sciences (CAS), Xiamen, China; ^3^School of Resources and Environmental Engineering, Anhui University, Hefei, China

**Keywords:** Feammox, Anammox bacteria, ammonium oxidation, iron(III) reduction, ^15^N_2_ production

## Abstract

Anaerobic ammonium oxidation coupled to nitrite reduction (termed as Anammox) was demonstrated as an efficient pathway to remove nitrogen from a wastewater treatment system. Recently, anaerobic ammonium oxidation was also identified to be linked to iron(III) reduction (termed Feammox) with dinitrogen, nitrite, or nitrate as end-product, reporting to enhance nitrogen removal from the wastewater treatment system. However, little is known about the role of Anammox bacteria in the Feammox process. Here, slurry from wastewater reactor amended with ferrihydrite was employed to investigate activity of Anammox bacteria in the Feammox process using the ^15^N isotopic tracing technique combined with 16S rRNA gene amplicon sequencing. A significantly positive relationship between rates of ^15^N_2_ production and iron(III) reduction indicated the occurrence of Feammox during incubation. Relative abundances of Anammox bacteria including *Brocadia*, *Kuenenia*, *Jettenia*, and unclassified Brocadiaceae were detected with low relative abundances, whereas Geobacteraceae dominated in the treatment throughout the incubation. ^15^N_2_ production rates significantly positively correlated with relative abundances of *Geobacter*, unclassified Geobacteraceae, and Anammox bacteria, revealing their contribution to nitrogen generation *via* Feammox. Overall, these findings suggested Anammox bacteria or cooperation between Anammox bacteria and iron(III) reducers serves a potential role in Feammox process.

## Introduction

Feammox [anaerobic ammonium oxidation coupled to iron(III) reduction] is a pathway of nitrogen cycling identified recently and makes a contribution to nitrogen loss in various environments, such as terrestrial (e.g., wetland, tropical rainforest, and paddy soils) and aquatic ecosystem (e.g., freshwater and marine) in addition to denitrification, co-denitrification, and anaerobic ammonium oxidation (Clement et al., 2005; Yang et al., 2012; Ding et al., 2014, 2017, 2019; Huang and Jaffé, 2014; Zhou et al., 2016). Previous studies showed that iron(III)-reducing bacteria such as *Anaeromyxobacter*, *Pseudomonas*, *Geobacter*, *Desulfosporosinus*, *Dechloromonas*, and *Geothrix* always dominated in the Feammox “pool” (Zhou et al., 2016; Li et al., 2019); however, these iron(III) reducers prefer to utilize organic carbon for iron(III) reduction. Only a minor part of iron(III) reduction (0.4–6.1%) by these bacteria is estimated to be associated with Feammox in the natural or artificial environments (Yang et al., 2012; Ding et al., 2014, 2017, 2019; Li et al., 2015; Zhou et al., 2016). As a replacement, ^15^NH_4_^+^ is generally employed to trace the occurrence of Feammox; however, it is hard to directly identify the Feammox microbes through DNA or RNA stable isotope probing because the Feammox process results in conversion of ^15^NH_4_^+^ to ^15^N_2_/^15^NO_3_^–^/^15^NO_2_^–^ but not assimilation of ^15^NH_4_^+^ into DNA and RNA. As a result, “Feammox microbes” are difficult to be directly captured from the incubation.

Sawayama (2006) has reported that the possibility of Feammox happened in a wastewater treatment system, which is recently expected to become another important way to remove nitrogen from the sludge of wastewater in addition to Anammox. Anammox is another anaerobic ammonium oxidation pathway that used nitrite as an electron acceptor with forming dinitrogen (Rikmann et al., 2014). Furthermore, the addition of iron(III) oxides is indicated to increase the efficiency of nitrogen removal in the system (Chen et al., 2014; Wang et al., 2016; Li H. et al., 2018; Yin et al., 2019). These discoveries further suggest the occurrence of Feammox in the Anammox-based nitrogen removal system of a wastewater treatment reactor. However, the Feammox-involving microorganisms in the wastewater treatment system is still under-characterized. Anammox bacteria, including members of the Planctomycetales such as *Brocadia*, *Kuenenia*, *Anammoxoglobus*, *Jettenia*, *Anammoximicrobium moscowii*, and *Scalindua* (Rikmann et al., 2014), possesses a versatile metabolism involved in the utilization of diverse electron donors (e.g., NH_4_^+^ and propionate) and acceptors (e.g., NO_2_^–^, NO_3_^–^, and SO_4_^2–^) (Strous et al., 2002; Cervantes et al., 2009; Rikmann et al., 2014; Rios-Del Toro and Cervantes, 2016; Rios-Del Toro et al., 2018). Inspired by these recent findings, we hypothesized that Fe(III) can play as terminal electron acceptors in anaerobic ammonium oxidation mediated by Anammox bacteria in the sludge from a wastewater treatment reactor.

In order to verify our hypothesis, sludge from a wastewater treatment reactor, which has been demonstrated to employ the Anammox process to remove nitrogen (Zhang et al., 2012), was used in this study. Through the ^15^NH_4_^+^-based isotopic tracing technique with 16S rRNA gene Illumina sequencing, we amended the sludge with ferrihydrite to investigate whether (1) Feammox occurred in the Anammox enrichment and (2) the Anammox organisms were involved in Feammox process.

## Materials and Methods

### Experimental Procedures

The sludge was obtained from a wastewater treatment reactor (Zhang et al., 2012). The dominant electron donor and acceptor were NH_4_^+^ and NO_2_^–^ in the wastewater treatment reactor, respectively, which has been established as a stable, completely autotrophic nitrogen removal process over nitrite (Canon). Theoretically, the Canon is a process combining partial nitrification with Anammox within a single reactor, which has been shown to be a cost-efficient autotrophic process for nitrogen removal, as it has no need for a carbon source and has low requirement for oxygen (Zhang et al., 2012). The characteristic of the sludge is detailed in Supplementary Table 1. The Feammox experiment was initiated by inoculating 10% (v/v) sludge into 20 ml basal medium and incubated at 30°C in the dark. The basal medium (pH 6.8–7.2) consists of MgCl_2_⋅6H_2_O (0.4 g L^–1^), CaCl_2_⋅H_2_O (0.1 g L^–1^), ^14^NH_4_Cl (0.027 g L^–1^), KH_2_PO_4_ (0.6 g L^–1^), 1 ml L^–1^ vitamin solution (Lovley and Phillips, 1988), 1 ml L^–1^ trace element solution (Lovley and Phillips, 1988), 30 mmol L^–1^ bicarbonate buffer, and 4 mmol L^–1^ Fe(III). The headspace of the serum vials was flushed with ultrapure helium. Ferrihydrite was synthesized as previously described (Kappler et al., 2014) and used as the Fe(III) source. The basal medium and ferrihydrite were autoclaved (120°C for 20 min) before use, and the vitamin solution and trace element solution were filtrated with a 0.22-μm filter from the stock solutions. In order to enrich the Feammox-associated microbial population, the cultures were anaerobically transferred (10%, v/v) to fresh medium for three generations once the Fe(III) was used up.

For the labeled experiment, ^14^NH_4_Cl was replaced by ^15^NH_4_Cl (^15^N, 99.14%; Cambridge Isotope Laboratories, Andover, MA, United States) to prepare the fresh medium. In brief, aliquots (2 ml) of the Feammox enrichment were centrifuged and washed three times with sterile deionized water before inoculating into the 20 ml of fresh ^15^NH_4_Cl-labeled medium. Three treatments were set up: (1) NH_4_^+^: Feammox enrichment was inoculated in the 20 ml of ^15^NH_4_Cl-added basal medium amended without ferrihydrite; (2) Fe(III): Feammox enrichment was inoculated in the 20 ml of ferrihydrite-containing basal medium amended without ^15^NH_4_Cl; and (3) Fe(III) + NH_4_^+^: Feammox enrichment was inoculated in the 20 ml of basal medium amended with both ferrihydrite and ^15^NH_4_Cl. The final concentrations of ^15^NH_4_Cl and ferrihydrite were 0.5 and 4 mmol L^–1^ in the treatments, respectively. The number of serum vials for each treatment was 4, 4, and 24, respectively. All the treatments were incubated at 30°C in a dark under anaerobic condition.

### Chemical Analysis

Ferrous iron and total iron were determined as described previously (Kappler et al., 2005). Briefly, Fe(II) was determined by anaerobically transferring 100 μl of culture suspension with a syringe into 900 μl of 40 mmol L^–1^ sulfamic acid and incubating for 1 h at room temperature. Total Fe was extracted using a mixture of 20 mmol L^–1^ hydroxylamine hydrochloride and 20 mmol L^–1^ sulfamic acid (v:v = 1:1) (Klueglein et al., 2015). A 100-μl extract was then added with 1 ml ferrozine solution (1 g ferrozine in 50 mmol L^–1^ HEPES buffer, pH 7) to generate the ferrous complex, which was quantified at 562 nm UV/Vis spectrometer. Change in Fe(II) concentrations between two given time points (23 days) were used to calculate iron(III) reduction rates.

For the ^15^N-N_2_ analysis, vials were shaken vigorously to equilibrate the dissolved phase with gaseous phase, and 1 ml of gas samples was collected from the headspace using gas-tight syringes and then injected into 12-ml glass vials (Exetainer; Labco, Lampeter, United Kingdom). The gas samples were taken on days 1, 4, 8, 10, 12, 14, 18, and 22, respectively. ^30^N_2_ and ^29^N_2_ concentrations were calculated by multiplying the moles of total N_2_ in the headspace by the ^30^N_2_ and ^29^N_2_ mole fractions (Zhou et al., 2016). The total N_2_ and N_2_O concentration in the headspace was measured using a robotized system coupled to a gas chromatograph (Agilent Technologies, Santa Clara, CA, United States) as previously described (Zhou et al., 2016). The mole fractions of ^30^N_2_ and ^29^N_2_ were determined by isotope ratio mass spectrometry (IRMS; Thermo Finnigan Delta V Advantage, Bremen, Germany) coupled with Gasbench II, respectively (Zhou et al., 2016). After gas collection, the remaining cultures were immediately centrifuged at 14,000 × *g* for 15 min and the pellets were used for DNA extraction. The resulting supernatant was filtered through 0.22-μm filters and then subjected to measurement of NH_4_^+^, NO_2_^–^, and NO_3_^–^ concentrations by ion chromatography (Dionex ICS-3000 system; Diones, Sunnyvale, CA, United States). All the liquid was sampled in the anaerobic glovebox (Shel Lab Bactron IV; Shel Lab, Cornelius, OR, United States) to avoid chemical oxidation. pH was analyzed using a dual-channel pH-ion-conductivity-dissolved oxygenmeter (X60; Thermo Fisher Scientific, Carlsbad, CA, United States) in the anaerobic glovebox.

### DNA Extraction and Illumina Sequencing

DNA was extracted using FastDNA Spin Kit (MP Biomedical, Illkirch-Graffenstaden, France) according to the manufacturer’s protocol and stored at −20°C for the molecular analyses. Since the biomass in the treatments amended with only Fe(III) or NH_4_^+^ was extremely low, there was enough DNA extracted from these treatments.

To investigate the bacterial community structures and compositions, the V4–V5 region of bacterial was amplified using the DNA extracted from the samples in the treatment of Fe(III) + NH_4_^+^ as template. The amplicons were purified, quantified, pooled, and then sequenced on an Illumina Miseq PE 250 platform (Novogene, Beijing, China) (Zhou et al., 2016). The forward primer was 515F (5′-GTGCCAGCMGCCGCGG-3′), and the reverse primer consisted of a 6-bp barcode and 907R (5′-CCGTCAATTCMTTTRAGTTT-3′) (Ren et al., 2014). Quantitative Insights into Microbial Ecology toolkit-version 1.9.0 (QIIME) was used to process and analyze sequences as previously described (Su et al., 2015). After removal of low-quality or ambiguous reads, operational taxonomic units (OTU) were determined at 97% similarity level using UCLUST clustering in accordance with the online instruction of QIIME for open-reference OTU pick, definition, and determination (Wang et al., 2007). The representative sequences of each OTU were assigned to taxonomy using an RDP classifier (Version 11).^1^

### Quantitative PCR

The abundance of relevant genes and microbial organisms, including bacterial 16S rRNA gene, *Geobacteraceae* spp., *Acidimicrobiaceae* spp. (the reported potential microbe responsible for Feammox) (Huang and Jaffé, 2014), *hzsB* (hydrazine synthase), *nirS* (nitrite reductase), and *nosZ* (nitrous oxide reductase) was analyzed with a real-time PCR Detection System (Roche 480; Roche, Indianapolis, IN, United States). The primer sets and thermal cycles were detailed in Supplementary Table 2. The 20-μl qPCR reaction contained 10 μl 2 × TransStart^®^ Top Green qPCR SuperMix (AQ131; Transgen Biotech, Beijing, China), 0.25 μM each primer, 0.8 μl bovine serum albumin (BSA, 20 mg ml^–1^), and 2 μl of fivefold diluted DNA as a template. The standard curve was obtained using 10-fold serial dilutions of plasmid DNA with target-genes. Three non-template controls were carried out for each quantitative assay. A melting curve for each reaction showed that only one special peak was detected. Only the reactions with efficiencies between 90 and 110%, and standard curves with correlation coefficient above 0.99 were employed in this study.

### Statistical Analyses

Analysis of variance (ANOVA) and Pearson correlation analysis were conducted by SPSS 18.0 (SPSS Inc., Chicago, IL, United States) and Origin 9.0 (OriginLab, Northampton, MA, United States). Statistical significance was performed using Duncan’s multiple range test and denoted at *p* < 0.05. The differences of the bacterial communities were analyzed by non-metric multidimensional scaling (NMDS) based on weighted UniFrac dissimilarity among samples, which was represented by the ordination axes (Tunney et al., 2013).

### Data Accessibility

The 16S rRNA gene sequences have been deposited in GenBank with accession number SRP116169.

## Results

### Iron(III) Reduction and Changes of N Species in the Enrichment

**FIGURE 1:**
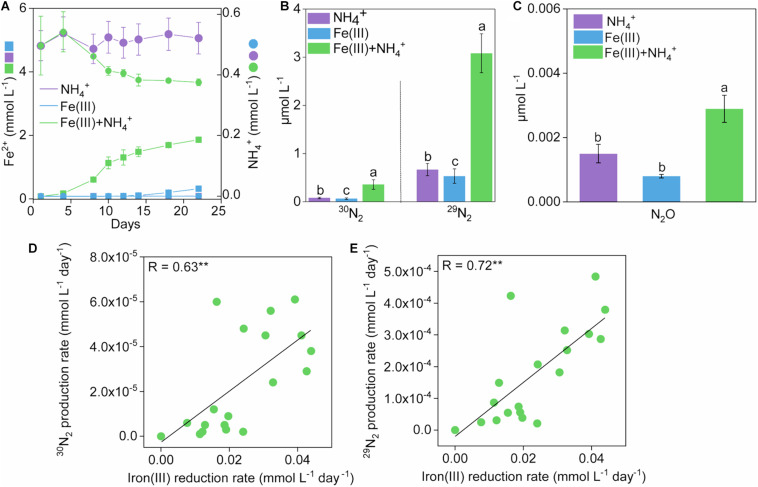
Development of Fe(II), NH_4_^+^ and N_2_ production in treatments during 22-day incubation. Kinetics of Fe(II) **(A)**, NH_4_^+^ **(B)**, and N_2_ **(C)** production in treatments of NH_4_^+^, Fe(III), and Fe(III) + NH_4_^+^ during 22-day incubation. Error bars represent standard deviations of three replications. The different lowercase letters above the error bar denote statistically significant (*p* < 0.05) differences among different treatments. **(D,E)** The correlation between rates of iron(III) reduction and ^15^N_2_ production in treatments of Fe(III) + NH_4_^+^ during 22-day incubation. “*” and “**” represent a statistically significant relationship between rates of iron(III) reduction and ^15^N_2_ production, which is denoted at *p* < 0.05 and *p* < 0.01, respectively.

In the Fe(III) + NH_4_^+^ treatment, Fe(II) increased up to 1.83 ± 0.010 mmol L^–1^ after 22-day anaerobic incubation (Figure 1A). In comparison, iron(III) reduction was not detected in the treatments only amended with Fe(III) or NH_4_^+^ after the 22-day incubation (Figure 1A).

Significant (*p* < 0.05) accumulation of ^30^N_2_ was detected in the Fe(III) + NH_4_^+^ treatment (0.36 μmol L^–1^) compared to that in the treatment of Fe(III) (0.064 μmol L^–1^) or NH_4_^+^ (0.080 μmol L^–1^) during the incubation (Figure 1B and Supplementary Figure 1). The ^29^N_2_ production rates showed similar trends to that of ^30^N_2_ (Figure 1B and Supplementary Figure 1). Headspace N_2_O exhibited a higher concentration in the treatment of Fe(III) + NH_4_^+^ (2.89 × 10^–3^ μmol L^–1^) than that in the treatment of Fe(III) (8.00 × 10^–4^ μmol L^–1^) or NH_4_^+^ (9.01 × 10^–4^ μmol L^–1^) during the incubation (Figure 1C). An amount of 0.12 mmol L^–1^ NH_4_^+^ was consumed in the treatment of Fe(III) + NH_4_^+^ (Figure 1A). Almost no utilization of NH_4_^+^ was observed in the NH_4_^+^ treatment (Figure 1A).

The rates of ^30^N_2_ and ^29^N_2_ production were significantly (*p* < 0.001) correlated with iron(III) reduction rates in the Fe(III) + NH_4_^+^ treatment (Figures 1D,E).

### Changes in Abundances of Bacteria and the N Cycling-Relevant Genes

**FIGURE 2:**
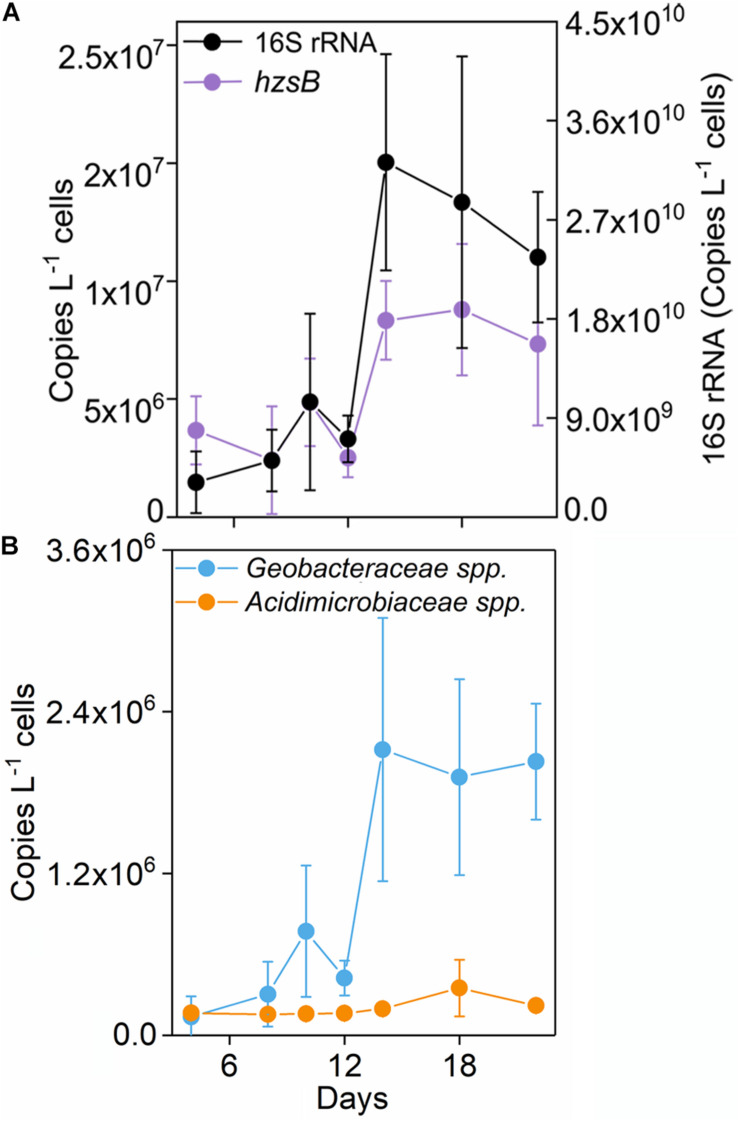
Copy numbers of 16S *rRNA* gene, and *nosZ* **(A)**, *Geobacteraceae* spp., and *Acidimicrobiaceae* spp. **(B)** in the treatment of Fe(III) + NH_4_^+^ during 22-day incubation. Error bars represent standard deviations of three replication.

16S rRNA gene copy number increased up to 2.36 × 10^10^ copies L^–1^ medium after incubation in the Fe(III) + NH_4_^+^ treatment (Figure 2A). Also, the abundances of *hzsB*, *nirS*, and *nosZ* rapidly elevated in the treatment of Fe(III) + NH_4_^+^ after 12 days (Figure 2 and Supplementary Figure 2). Especially, the gene copy numbers were higher for the genes *nirS* (1.26 × 10^7^) and *nosZ* (2.62 × 10^7^) than that for the gene *hzsB* (7.36 × 10^6^ copies L^–1^ medium) in the treatment of Fe(III) + NH_4_^+^ (Figure 2A).

The abundance of *Geobacteraceae* spp. was about 2.03 × 10^6^ copies L^–1^ medium after 22-day incubation in the treatment of Fe(III) + NH_4_^+^ (Figure 2B). By contrast, *Acidimicrobiaceae* spp. kept constantly low abundance throughout the incubation (Figure 2B).

### Shift of Bacterial Community Composition

**FIGURE 3:**
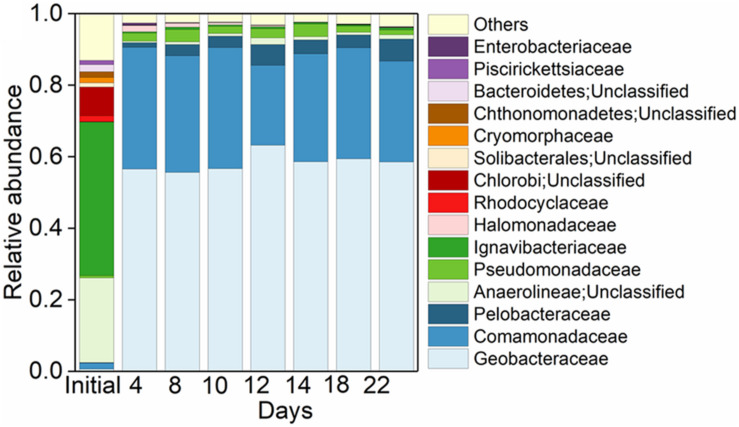
Pattern of microbial community in treatments. Top 15 taxa at family level in the initial Anammox inoculant and the Anammox inoculant-based treatment of Fe(III) + NH_4_^+^ during the 22-day incubation.

In the initial inoculant, 42.94% of the total bacterial community were affiliated to the family of Ignavibacteriaceae, followed by unclassified Anaerolineae (23.68%) and unclassified Chlorobi (8.02%) (Figure 3). While in the treatment of Fe(III) + NH_4_^+^, Geobacteraceae was the family with the highest relative abundance, followed by Comamonadaceae, Pelobacteraceae, and Pseudomonadaceae (Figure 3). These four families occupied up to 96.13% of the total microbial community (Figure 3).

### Shift in the Relative Abundances of Iron(III)-Reducers and Anammox Bacteria

**FIGURE 4:**
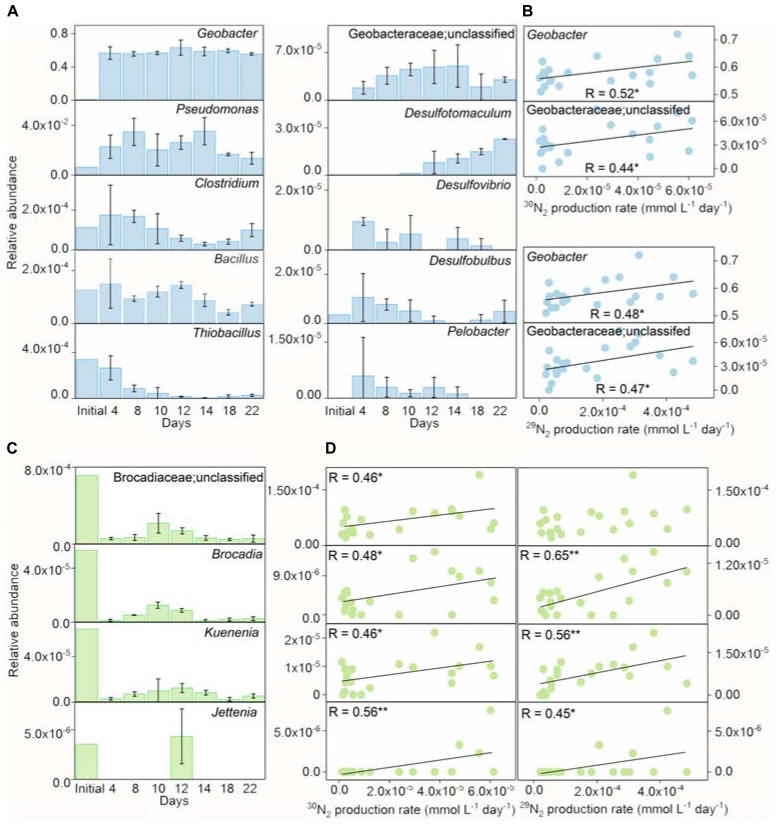
The relative abundances of iron(III) reducers and Anammox bacteria during incubation. The relative abundances of iron(III) reducers **(A)** and the correlation between the relative abundances of *Geobacter*/unclassified Geobacteraceae and ^15^N_2_ production rates **(B)** in the inoculant-based treatment of Fe(III) + NH_4_^+^ during 22-day incubation. Error bars represent standard deviations of three replications. The relative abundances of Brocadiaceae (unclassified), *Brocadia*, *Kuenenia*, and *Jettenia* **(C)**, and the correlation between the relative abundance of *Geobacter* and ^15^N_2_ production rates **(D)** in the inoculant-based treatment of Fe(III) + NH_4_^+^. Error bars represent standard deviations of three replication. “*” and “**” represent a statistically significant relationship between relative abundances of iron(III) reducers, Anammox bacteria, and ^15^N_2_ production, which is denoted at *p* < 0.05 and *p* < 0.01, respectively.

The detected iron(III) reducers included *Geobacter*, *Pseudomonas*, *Clostridium*, *Bacillus*, *Thiobacillus*, unclassified *Geobacteraceae*, *Desulfotomaculum*, *Desulfovibrio*, *Desulfobulbus*, and *Pelobacter* in the treatment of Fe(III) + NH_4_^+^ (Figure 4A). In comparison with the initial inoculant, the genera of *Geobacter*, *Pseudomonas*, *Clostridium*, *Desulfovibrio*, Desulfotomaculum, and *Pelobacter* were significantly enriched, while the relative abundance of *Bacillus* and *Thiobacillus* decreased in the treatment of Fe(III) + NH_4_^+^ during the incubation (Figure 4A). Of all the iron(III) reducers, the change in relative abundances of *Geobacter* and unclassified Geobacteraceae were significantly (*p* < 0.05) correlated with the ^30^N_2_ and ^29^N_2_ production rates in the treatment of Fe(III) + NH_4_^+^ (Figure 4B).

The Anammox-relevant Planctomycetes remained with low relative abundances in the treatment of Fe(III) + NH_4_^+^ after 22-day incubation. Dynamics of relative abundances of Anammox-relevant taxa, including unclassified Brocadiaceae, *Brocadia*, *Kuenenia*, and *Jettenia*, displayed a similar pattern in the treatment of Fe(III) + NH_4_^+^. These relative abundances of Anammox bacteria reached a peak on days 10–12 and then decreased during the incubation (Figure 4C). The relative abundances of these Anammox bacteria significantly (*p* < 0.05) correlated with the rates of ^30^N_2_ and ^29^N_2_ production (Figure 4D).

## Discussion

Feammox is a recently identified pathway of dinitrogen generation, expecting to be applied to remove nitrogen from a wastewater treatment system. In this study, we aimed to verify the occurrence of Feammox and investigate the Feammox-associated microbes in the sludge of a wastewater treatment system. Detection of ^15^N_2_ production from sludge amended with iron(III) indicated the existence of Feammox in the incubation. Anammox bacteria such as Brocadiaceae, *Kuenenia*, and *Jettenia* and iron(III) reducers including *Geobacter* and unclassified Geobacteraceae were found potentially involved in the Feammox process.

The Feammox incubation was established using the sludge as inoculant, which was subjected to three generations with freshly prepared medium under anaerobic condition. As a result, the major electron acceptor for anaerobic oxidation of ^15^N-NH_4_^+^ was ferrihydrite during the incubation. Previous reports suggested that Anammox can be linked to the microbial reduction of natural organic matters (Rios-Del Toro et al., 2018), likely originating from the breakdown of dead biomass, for example, carbohydrate residues including cellulose and lignin (Siemann et al., 2012). The transformation between oxidized and reduced state enables the quinone group-abundant organic matters serving as electron shuttles to mediate anaerobic oxidation of ammonium to N_2_ (Westereng et al., 2015; Rios-Del Toro et al., 2018). However, the standard Gibbs free energy released from this process (NH_4_^+^ + 1.5 quinone-NOM_ox_ → 0.5N_2_ + 1.5 quinoneH_2_-NOM_red_ + 4H^+^) ranged from 5.8 kJ mol^–1^ to −124.6 kJ mol^–1^ (Rios-Del Toro et al., 2018), greatly lower than that produced from the Feammox process [3Fe(OH)_3_ + 5H^+^ + NH_4_^+^ → 3Fe^2+^ + 9H_2_O + 0.5N_2_, Δ_r_G_m_ = −245 kJ mol^–1^; 6Fe(OH)_3_ + 10H^+^ + NH_4_^+^ → 6Fe^2+^ + 16H_2_O + NO_2_^–^, Δ_r_G_m_ = −164 kJ mol^–1^; 8Fe(OH)_3_ + 14H^+^ + NH_4_^+^ → 8Fe^2+^ + 21H_2_O + NO_3_^–^, Δ_r_G_m_ = −207 kJ mol^–1^] (Yang et al., 2012). Therefore, Anammox was probably coupled to ferrihydrite reduction during the incubation. The significant accumulation of ^30^N_2_ provided a solid evidence for the occurrence of Feammox in the treatment of Fe(III) + NH_4_^+^. Codenitrification is another potential source of ^30^N_2_ (Laughlin and Stevens, 2002). However, it can be ruled out in this study because other ^15^N-labeled nitrogen compounds (e.g., hydrazine and amino compounds) that could reduce ^15^NO_2_^–^ and ^15^NO_3_^–^ to N_2_ were not available in the culture. Under these conditions, direct N_2_ production from Feammox, or Feammox-produced NO_2_^–^ or NO_3_^–^ followed by denitrification or Anammox are the possible pathways for ^30^N_2_ generation, supporting the occurrence of Feammox in the treatment of Fe(III) + NH_4_^+^. The positive correlation (*p* < 0.0001; Figures 1D,E) between Fe(III) reduction and ^30^N_2_/^29^N_2_ production rates further verified the existence of Feammox in the treatment of Fe(III) + NH_4_^+^ during the anoxic incubation.

The amount of ^15^N_2_, N_2_O, and ^15^NO_x_^–^ (3.63 μmol L^–1^; Supplementary Table 3) produced from the treatment of Fe(III) + NH_4_^+^ was far less than that of NH_4_^+^ depleted during the incubation, indicating that the majority of NH_4_^+^ was assimilated into microbial biomass (Tupas and Koike, 1990). The abundant genera such as *Pseudomonas* and *Bacillus* detected in this study, which are able to assimilate ammonia during their growth (Kim and Hollocher, 1982; Kanamori et al., 1989), may be the major NH_4_^+^ consumers in the treatment of Fe(III) + NH_4_^+^.

The molar ratio of reduced Fe (III) to the total NH_4_^+^ oxidation was about 15.41 in the Fe(III) + NH_4_^+^ treatment (Figure 1), which did not match the stoichiometry (ranging from 3 to 8) in the three Feammox equations (Yang et al., 2012; Ding et al., 2014; Zhou et al., 2016). This suggested that only a minor fraction of reduced Fe(III) was linked to the NH_4_^+^ oxidation in the Fe(III) + NH_4_^+^ treatment. According to the thermodynamic calculations, the amount of iron reduction associated with Feammox was 0.36–0.96 mmol L^–1^, accounting for 19.67–52.46% of total Fe(III) reduction in the treatment of Fe(III) + NH_4_^+^. Thus, a majority of the reduced Fe(III) was linked to the oxidation of other substrates mediated by microorganisms. Because exogenous organic matter was not added in the treatment of Fe(III) + NH_4_^+^, the substrates might be organic compounds sourced from the dead biomass or extracellular secretion from the microbes. Among all the iron(III)-reducing bacteria, the family of Geobacteraceae was abundant in the treatment of Fe(III) + NH_4_^+^ (Figure 3). The genus of *Geobacter* is able to reduce Fe(III) associated with organic substrate oxidation to support its growth (Lovley, 1991). The relative abundance of Geobacteraceae showed an increase on days 10 and 12, which was in agreement with the rapidly increasing copy number of *Geobacter* after 12-day incubation in the treatment of Fe(III) + NH_4_^+^ (Figures 2B, 3, 4A), indicating *Geobacter* was the dominant genus in Geobacteraceae. The positive correlation between the abundance of *Geobacter* and accumulation of ^15^N_2_ in the treatment of Fe(III) + NH_4_^+^ (Figure 4B) suggested that *Geobacter* may exert a potential role in Feammox.

**FIGURE 5:**
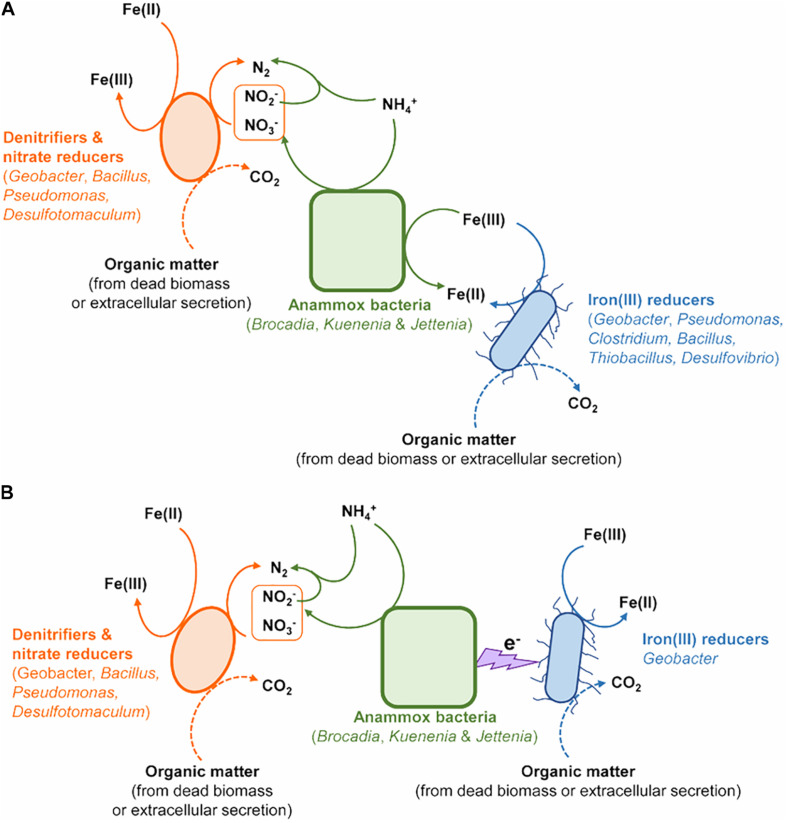
The potential models of microorganisms contributing to N removal *via* Feammox. **(A)** Anammox bacteria directly involved in the Feammox-associated N removal. **(B)** Interaction between Anammox bacteria and *Geobacter* linked to Feammox-associated N removal.

The abundance of Anammox bacteria, including Brocadiaceae, *Kuenenia*, and *Jettenia*, showed significant correlation with ^15^N_2_ production in the treatment of Fe(III) + NH_4_^+^ (Figure 4D), suggesting Anammox bacteria were linked with Feammox during the incubation. Although the mechanism about the role of Anammox bacteria as Feammox players was still unknown, a variety of reports have shown their versatile metabolism. Firstly, Anammox bacteria are capable of anaerobically oxidizing ammonium *via* anammoxosome coupled with other electron acceptors such as sulfate in addition to nitrite (Liu et al., 2008; Rikmann et al., 2014). The ΔG_o_ of sulfate-reducing anaerobic ammonium oxidation proceeded by Anammox species *Anammoxoglobus sulfate* (2NH_4_^+^ + SO_4_^2–^ → S_o_ + N_2_ + 4H_2_O ΔG_o_ = −46 kJ mol^–1^; 8NH_4_^+^ + 3SO_4_^2–^ → HS^–^ + 4N_2_ + 12H_2_O + 5H^+^ ΔG_o_ = −22 kJ mol^–1^) is obviously lower than that of Feammox (Liu et al., 2008; Rikmann et al., 2014), suggesting that Anammox bacteria in the enrichment should favor Feammox. Secondly, genera of *Brocadia* and *Kuenenia* have iron(III)-reducing ability using organic matter (e.g., formate, acetate, and propionate) as electron donor (Graaf et al., 1996; Zhao et al., 2014); 80% of the ferric iron reductase in these Anammox bacteria locates in the membrane fraction and part of them termed as dissimilatory ferric iron reductases are the essential terminal reductase of the Fe(III) respiratory pathway in iron(III)-reducing bacteria (Schröder et al., 2003; Zhao et al., 2014). It provided a cue that the Anammox bacteria detected in our study, including Brocadiaceae, *Kuenenia*, and *Jettenia*, were capable of reducing iron(III) linked to oxidizing ammonium anaerobically at same time. Moreover, several publications demonstrated the potential role of Anammox bacteria in Feammox based on the increase in the N_2_ production after amendment with iron(III) oxides in the Anammox sludge (Chen et al., 2014; Wang et al., 2016; Li H. et al., 2018; Li X. et al., 2018; Yin et al., 2019). The Feammox bacteria *Acidimicrobiaceae* sp. were previously identified in acid soil with pH between 3.5 and 4.5 (Huang and Jaffé, 2014); however, the gene copy of the *Acidimicrobiaceae* spp. was extremely low (Figure 2B), and members of this family were not detected *via* Illumina sequencing in this study, which might indicate that this reported family made very little contribution to Feammox-linked N_2_ production under neutral condition. In addition, the aerobic ammonium-oxidizing bacteria *Nitrosomonas* spp. were found to be highly enriched in the ammonium-containing anoxic condition (Lek Noophan et al., 2009), whereas they were not detected through Illumina sequencing and *amoA*-based qPCR (data not shown) in the treatment of Fe(III) + NH_4_^+^ in this study. All of these disclosed that the Anammox bacteria Brocadiaceae, *Kuenenia*, and *Jettenia* had potential for Feammox-associated anaerobic ammonium oxidation (Figure 5A).

Cooperation between Anammox bacteria and iron(III)-reducers such as *Geobacter* could also complete the Feammox process (Figure 5B). The conductive pili of *Geobacter* provide the chance to extend electron transfer ability beyond the outer surface of their cells (Reguera et al., 2005). These pili offer the possibility for *Geobacter* to accept electrons from anaerobic ammonium oxidation by Anammox bacteria through periplasmic or outer membrane electron transfer protein (Reguera et al., 2005). The increase in the gene copy numbers of *nirS*, *nosZ*, *hzsB*, and relative abundances in nitrate reducers/denitrifiers (including *Geobacter*, *Pseudomonas*, *Bacillus*, *Clostridium*, and *Desulfotomaculum*) (Zhou et al., 2019) further indicated the contribution of denitrification, nitrate reduction, Anammox and nitrate reduction dependent iron(II) oxidation to N turnover in the Fe(III) + NH_4_^+^ treatment during the incubation (Figures 5A,B).

Potential Feammox rate was estimated with a value of 0.49 μg N kg^–1^ d^–1^ based on the ^30^N_2_ production rates, which was comparable to that reported in paddy soil (0.17–0.59 μg N kg^–1^ d^–1^), intertidal wetland (0.24–0.36 μg N kg^–1^ d^–1^), and tropical forest soil (about 0.32 mg N kg^–1^ d^–1^) (Clement et al., 2005; Yang et al., 2012; Ding et al., 2014; Li et al., 2019). However, the contribution of Feammox to N loss was much lower than that of Anammox enrichment from the sludge (4.1 × 10^6^ μg N kg^–1^ d^–1^) (Chen et al., 2014), suggesting a substantially low N removal efficiency *via* Feammox. Furthermore, the minor contribution ratio of Feammox to N loss in this study was inconsistent with the previous reports in the Anammox sludge, which showed that Fe(III) addition increased the N removal up to 0.8 × 10^6^ μg N kg^–1^ d^–1^ (Chen et al., 2014; Wang et al., 2016). The significantly lower relative abundance of Anammox bacteria in the treatment of Fe(III) + NH_4_^+^ (Figures 2A, 3, 4C) than the those reported in the previous wastewater treatment system (Chen et al., 2014; Wang et al., 2016) may be an important reason for low amount of N_2_ *via* Feammox. Firstly, limited capability of substrate utilization by Anammox bacteria led to slow growth rate and long doubling time (Jetten et al., 2005). As a result, these Anammox bacteria were overwhelmed by iron(III) reducers that could efficiently obtain energy from dissimilatory iron(III) reduction. Secondly, the relative abundances of the Brocadiaceae, *Kuenenia*, and *Jettenia* decreased in the treatment of Fe(III) + NH_4_^+^ (Figure 4C), which was likely due to the accumulation of NO_2_^–^/NO_3_^–^ after 12-day incubation (Supplementary Table 3). Iron(II) coexistence with NO_2_^–^ (Supplementary Figure 2), which might lead to the NO_x_^–^ dependent Fe(II) oxidation, can severely inhibit the activity of Anammox bacteria (Zhao et al., 2014). Thirdly, dilution of Anammox bacteria *via* three generations of subculture may be another important reason for the low activity of Feammox-associated N_2_ production decreased during the incubation. Besides, Fe_3_O_4_ can form from rapid iron(III) reduction with the production of Fe(II) absorbed on the ferric oxides surface and then improve N removal during Feammox process (Kappler et al., 2014; Li H. et al., 2018; Li X. et al., 2018). Hence, it can be inferred that Fe(II) was accumulated with relatively low extent after subculturing in the treatment of Fe(III) + NH_4_^+^; therefore, the amount of Fe_3_O_4_ produced in the treatment was lower compared to the sludge reactor in continuous operation.

## Conclusion

This study demonstrated the occurrence of Feammox in Anammox inoculant-based enrichment. The relative abundances of *Geobacter* and Anammox bacteria such as Brocadiaceae, *Kuenenia*, and *Jettenia* were significantly correlated with ^15^N_2_ production rates, indicating their potential role in Feammox-involved N removal. We proposed that sole Anammox bacteria or cooperation between Anammox bacteria and *Geobacter* or unclassified Geobacteraceae could complete the Feammox process during the incubation. Our results suggested the potential role of Anammox bacteria in the nitrogen removal *via* the Feammox process.

## Data Availability Statement

The datasets presented in this study can be found in online repositories. The names of the repository/repositories and accession number(s) can be found in the article/Supplementary Material.

## Author Contributions

X-RY, HL, and G-WZ did the experiments, conceived and designed the project, and analyzed the data. J-QS gave assistance in lab work and laboratory analyses. X-RY wrote the manuscript. X-RY, HL, J-QS, and G-WZ revised the manuscript. All authors read and approved the final manuscript.

## Conflict of Interest

The authors declare that the research was conducted in the absence of any commercial or financial relationships that could be construed as a potential conflict of interest.

## Publisher’s Note

All claims expressed in this article are solely those of the authors and do not necessarily represent those of their affiliated organizations, or those of the publisher, the editors and the reviewers. Any product that may be evaluated in this article, or claim that may be made by its manufacturer, is not guaranteed or endorsed by the publisher.

## Footnotes

1
http://rdp.cme.msu.edu

## Supplementary Material

Supplementary Figure 1 The dynamics of ^29^N_2_ and ^30^N_2_ production in the treatment of Fe(III) + NH_4_^+^ during 22-day incubation.

Supplementary Figure 2 The time course of abundances of *nirS* and *nosZ*, and concentrations of NO_3_^–^ and NO_2_^–^ in the treatment of Fe(III) + NH_4_^+^ during 22-day incubation.

Supplementary Table 1 Characteristic of the initial inoculant slurry.

Supplementary Table 2 Primers and qPCR processes used in this study.

Supplementary Table 3 The concentrations of ^15^NO_x_^–^ in the Fe(III) + NH_4_^+^ treatment after 22-day incubation.
