# Supplementary material for: Anammox Bacteria Are Potentially Involved in Anaerobic Ammonium Oxidation Coupled to Iron(III) Reduction in the Wastewater Treatment System
Source: Front Microbiol. 2021 Sep 10;12:717249. doi: 10.3389/fmicb.2021.717249 (PMC8461334; doi:10.3389/fmicb.2021.717249)
Supplement: Supplementary Figure 1 — The dynamics of 29N2 and 30N2 production in the treatment of Fe(III) + NH4+ during 22-day incubation. [file Data_Sheet_1.docx]

**Supporting Material**

**Anammox bacteria are potentiallyinvolved in anaerobic ammonium oxidation coupled to iron(III) reduction in the waste water treatment system**

Xiao-Ru Yang ^1,2^,Hu Li^1,2^, Jian-Qiang Su^1,2^, and Guo-Wei Zhou^1,2,3*^

1.Key Lab of Urban Environment and Health, Institute of Urban Environment, Chinese Academy of Sciences, Xiamen 361021, People’s Republic of China

2.Center for Excellence in Regional Atmospheric Environment, Institute of Urban Environment, Chinese Academy of Sciences, Xiamen 361021, People’s Republic of China

3. School of Resources and Environmental Engineering, Anhui University, Hefei, 230601, People’s Republic of China

Address correspondence to Guo-Wei Zhou, [gwzhou@ahu.edu.cn](mailto:gwzhou@ahu.edu.cn).

Phone: +86-551- 63861441

Fax: +86-551- 63861441

**Materials and methods**

**δ^15^N Determination of nitrate and nitrite**

δ^15^N of NO_3_^-^was determined based on the following two equations as described before (Zhao et al., 2019). In brief, after liquid sample taken during the incubation, NaCl was amended to the samples in centrifuge tubes, and imidazole (1 M) and HCl (0.5 M) were added to the solution to adjust the pH (pH = 8),and then 0.3 g of Cadmium(Cd) was added under the anoxic condition. Then, thecentrifuge tubeswereshaken at 120 rpm (37 °C). After precipitation, solution was separated from Cd, and 4 mL of the upper layer solution was moved to a 12-mL headspace vial. 20% acetic acid solution was mixed with NaN_3_solution (2 M) (v : v = 1 : 1) in a fume hood, and then purged with helium gas (99%) at a flow rate of 70 mL min^–1^ for 10 min toexpel the possible remining N_2_. The purged sodium azide acetate solution (0.2 mL) was thentransferred into a 12mL headspace vial, and the reaction wasceased by addition of NaOH solution (0.4 mL, 10 M) after inverting the buckle. δ^15^N were determined by anautosampler with N_2_O gas in the headspace of bottle under the Precon+Gas Bench II system (Thermo Scientific, Bremen, Germany). Without addition of Cd, determination of δ^15^N- NO_2_^-^was same with that of δ^15^N- NO_3_^-^based on equations (1), (2.1), (2.2)and (2.3)(Zhao et al., 2019). Finally, the concentrations of ^15^N-NO_2_^-^ and ^15^N-NO_3_^-^were calculated as the product of NO_2_^-^ and NO_3_^-^.

NO_3_^–^ + Cd + H_2_O → NO_2_^–^ + Cd^2+^ + 2OH^–^ (1)(Wang et al., 2015)

NO_2_^–^ + H^+^→ HNO_2_ (2.1)(Mcilvin and Altabet 2005)

HNO_2_ + H_2_O → H_2_NO_2_+ + OH^–^(2.2)(Mcilvin and Altabet 2005)

H_2_NO_2_^+^ + N_3_^–^→ N_3_NO + H_2_O (slow) → N_2_O + N_2_(fast) (2.3)(Mcilvin and Altabet 2005)


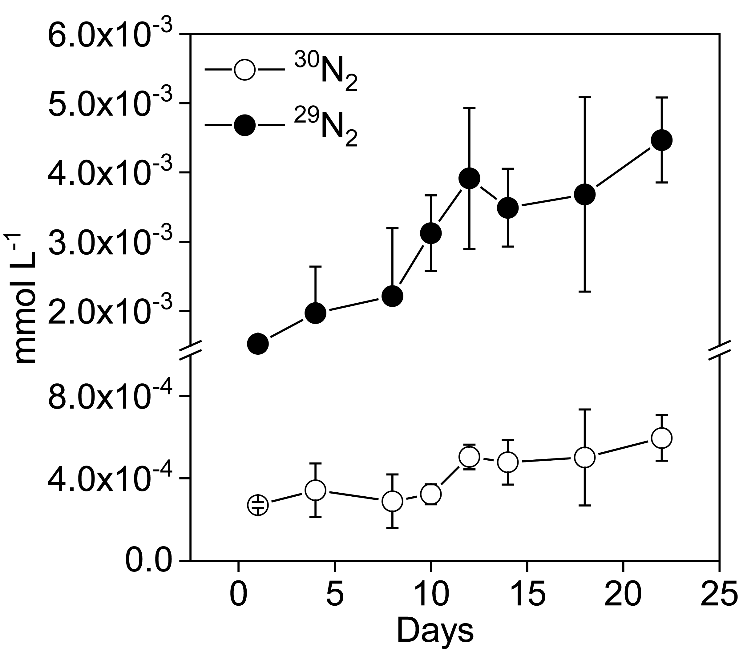


**Supplementary Figure 1**. The dynamics of ^29^N_2_ and ^30^N_2_production in the treatment of Fe(III) + NH_4_^+^ during22-day incubation.Error bars represent standard deviations of three replication.


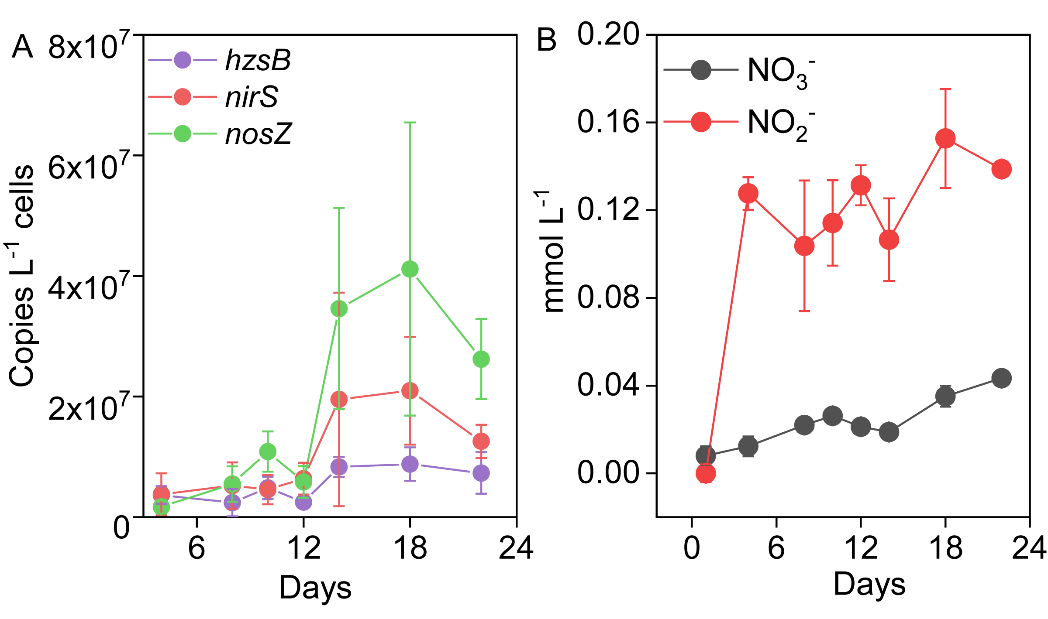


**Supplementary Figure 2**. The time course of abundances of *nirS* and *nosZ*, and concentrations of NO_3_^-^ and NO_2_^-^in the treatment of Fe(III) + NH_4_^+^ during 22-day incubation.Error bars represent standard deviations of three replication.

**Supplementary Table1**. Characteristic of thesludgefrom the waste-water treatment reactor(Zhang et al., 2012).

| Items | NH_4_^+^-N | NO_2_^-^-N | NO_3_^-^-N | Fe^2+^ |
| --- | --- | --- | --- | --- |
| Concentration  (mg·L^-1^) | 75.17 | 77.02 | 4.78 | < 1.00 |

**Supplementary Table 2**.Primers and qPCR processes used in this study.

| **Target-genes** | **Function** |  | **Primers(sequences 5’-3’)** | **qPCR processes** | **References** |
| --- | --- | --- | --- | --- | --- |
| 16S rRNA | Characterization of diversity and abundance of bacteria | 515R  907R | GTGCCAGCMGCCGCGG  CCGTCAATTCMTTTRAGTTT | 5 min at 95^o^C, followed by 30 cycles  of 95^o^C 15s, 60^o^C 1min and 72^o^C 15s | (Lane et al., 1985) |
| *Geobacteraceae* | The reported potential microbe responsible for Feammox | 577F  822R | GCGTGTAGGCGGTTTSTTAA  TACCCGCRACACCTAGTACT | 2 min at 95^o^C, followed by 30 cycles  of 95^o^C 15s, 55^o^C 30s and 72^o^C 30s | (Azizian et al., 2010) |
| *Acidimicrobiaceae* | The reported potential microbe responsible for Feammox | 342F  439R | GCA ATGGGGGAA ACCCTGAC  ACCGTCAATTTCGTCCCTGC | 2 min at 95^o^C, followed by 30 cycles  of 95^o^C 15s, 58^o^C 30s and 72^o^C 30s | (Huang and Jaffé 2014) |
| *nirS* | Nitrite reductase | *nirS*-cd3aF  *nirS*-R3cd | GTSAACGTSAAGGARACSGG  GASTTCGGRTGSGTCTTGA | 2 min at 95^o^C, followed by 30 cycles  of 95^o^C 5s, 62^o^C 40s and 72^o^C 35s | (Throbäck et al., 2004) |
| *nosZ* | Nitrous oxide reductase | *nosZ*-F  *nosZ*-1622R | CGYTGTTCMTCGACAGCCAG  CGSACCTTSTTGCCSTYGCG | 1 min at 95^o^C, followed by 30 cycles  of 95^o^C 15s, 60^o^C 15s and 72^o^C 34s | (Throbäck et al., 2004) |
| *hzsB* | Hydrazine synthase | *hzsB*-396F  *hzsB*-742R | ARGGHTGGGGHAGYTGGAAG  GTYCCHACRTCATGVGTCTG | 3 min at 95^o^C, followed by 30 cycles  of 95^o^C 30s, 59^o^C 30s and 72^o^C 30s | (Wang et al., 2012) |
| Archaeal *amoA* | Archael ammonia monooxygenase | *amoA*F  *amoA*R | STAATGGTCTGGCTTAGAC  GCGGCCATCCATCTGTATGT | 5 min at 94^o^C, followed by 30 cycles  of 94^o^C 45s, 58^o^C 60s and 72^o^C 60s | (Francis et al., 2005) |
| Bacterial *amoA* | Bacterial ammonia monooxygenase | *amoA*1F  *amoA*2R | GGGGTTTCTACTGGTGGT  CCCCTCKGSAAAGCCTTCTTC | 2 min at 95^o^C, followed by 30 cycles  of 95^o^C 15s, 56^o^C 30s and 72^o^C 30s | (Rotthauwe et al., 1997) |

**Supplementary Table 3.**The concentrations of ^15^NO_x_^-^ in the Fe(III) + NH_4_^+^ treatment after 22-day incubation.

| ^15^NO_x_^-^ | Concentration (μmol L^-1^) |
| --- | --- |
| ^15^NO_2_^-^ | 2.64 ± 0.13 |
| ^15^NO_3_^-^ | 0.99 ± 0.083 |

**References**

Azizian, M.F., Marshall, I.P., Behrens, S., Spormann, A.M., Semprini, L. 2010. Comparison of lactate, formate, and propionate as hydrogen donors for the reductive dehalogenation of trichloroethene in a continuous-flow column. J. Contam. Hydrol. 113 (1-4), 77-92. doi: 10.1016/j.jconhyd.2010.02.004.

Francis, C.A., Roberts, K.J., Beman, J.M., Santoro, A.E., Oakley, B.B. 2005. Ubiquity and diversity of ammonia-oxidizing archaea in water columns and sediments of the ocean. Proc. Natl. Acad. Sci. U. S. A. 102 (41), 14683-14688.

Huang, S., Jaffé, P.R. 2014. Characterization of incubation experiments and development of an enrichment culture capable of ammonium oxidation under iron reducing conditions. Biogeosciences 11 (8), 12295-12321. doi: 10.5194/bgd-11-12295-2014.

Lane, D.J., Pace, B., Olsen, G.J., Stahl, D.A., Sogin, M.L., Pace, N.R. 1985. Rapid determination of 16S ribosomal RNA sequences for phylogenetic analyses. Proc. Natl. Acad. Sci. U.S.A 82 (20), 6955-6959.

Mcilvin, M.R., Altabet, M.A. 2005. Chemical conversion of nitrate and nitrite to nitrous oxide for nitrogen and oxygen isotopic analysis in freshwater and seawater. Anal. Chem. 77 (17), 5589-5595.

Rotthauwe, J.H., Witzel, K.P., Liesack, W. 1997. The ammonia monooxygenase structural gene *amoA* as a functional marker: molecular fine-scale analysis of natural ammonia-oxidizing populations. Appl. Environ. Microbiol. 63 (12), 4704.

Throbäck, I.N., Enwall, K., Jarvis, Å., Hallin, S. 2004. Reassessing PCR primers targeting *nirS*, *nirK* and *nosZ* genes for community surveys of denitrifying bacteria with DGGE. FEMS Microbiol. Ecol. 49 (3), 401-417. doi: 10.1016/j.femsec.2004.04.011.

Wang, X., Cao, Y.C., Han, Y., Tang, H.Y., Wang, R.H., Sun, X.L., Sun, Y.F. 2015. Determination of nitrogen and oxygen isotope ratio of nitrate in water. Acta Pedologica Sinica 52 (3), 558-566. doi: 10.11766/trxb201405080224.

Wang, Y., Zhu, G.B., Harhangi, H.R., Zhu, B.l., Jetten, M.S., Yin, C.Q., Op den Camp, H.J. 2012. Co-occurrence and distribution of nitrite-dependent anaerobic ammonium and methane-oxidizing bacteria in a paddy soil. FEMS Microbiol. Lett. 336 (2), 79-88. doi: 10.1111/j.1574-6968.2012.02654.x.

Zhang, Z.J., Li, Y., Chen, S., Wang, S., Bao, X. 2012. Simultaneous nitrogen and carbon removal from swine digester liquor by the Canon process and denitrification. Bioresour. Technol. 114 84-89. doi: 10.1016/j.biortech.2012.03.006.

Zhao, Z.Y., Cao, F., Zhang, W.Q., Zhai, X.Y., Fang, Y., Fan, M.Y., Zhang, Y.L. 2019. Determination of stable nitrogen and oxygen isotope ratios in atmospheric aerosol nitrates. Chinese J. Anal. Chem. 47 (6), 907-915. doi: 10.1016/s1872-2040(19)61166-7.
